# Current advancements of modelling schizophrenia using patient-derived induced pluripotent stem cells

**DOI:** 10.1186/s40478-022-01460-2

**Published:** 2022-12-16

**Authors:** Ugne Dubonyte, Andrea Asenjo-Martinez, Thomas Werge, Kasper Lage, Agnete Kirkeby

**Affiliations:** 1 Department of Neuroscience and Novo Nordisk Foundation Center for Stem Cell Medicine (reNEW), University of Copenhagen, Copenhagen, Denmark; 2 Institute of Biological Psychiatry, Mental Health Services, Copenhagen University Hospital, Copenhagen, Denmark; 3 Department of Clinical Medicine and Lundbeck Foundation Center for GeoGenetics, GLOBE Institute, University of Copenhagen, Copenhagen, Denmark; 4 Stanley Center for Psychiatric Research and The Novo Nordisk Foundation Center for Genomic Mechanisms of Disease, Broad Institute of MIT and Harvard, Cambridge, MA USA; 5 Department of Surgery, Massachusetts General Hospital, Boston, MA USA; 6 Department of Experimental Medical Science and Wallenberg Center for Molecular Medicine, Lund University, Lund, Sweden

## Abstract

Schizophrenia (SZ) is a severe psychiatric disorder, with a prevalence of 1–2% world-wide and substantial health- and social care costs. The pathology is influenced by both genetic and environmental factors, however the underlying cause still remains elusive. SZ has symptoms including delusions, hallucinations, confused thoughts, diminished emotional responses, social withdrawal and anhedonia. The onset of psychosis is usually in late adolescence or early adulthood. Multiple genome-wide association and whole exome sequencing studies have provided extraordinary insights into the genetic variants underlying familial as well as polygenic forms of the disease. Nonetheless, a major limitation in schizophrenia research remains the lack of clinically relevant animal models, which in turn hampers the development of novel effective therapies for the patients. The emergence of human induced pluripotent stem cell (hiPSC) technology has allowed researchers to work with SZ patient-derived neuronal and glial cell types in vitro and to investigate the molecular basis of the disorder in a human neuronal context. In this review, we summarise findings from available studies using hiPSC-based neural models and discuss how these have provided new insights into molecular and cellular pathways of SZ. Further, we highlight different examples of how these models have shown alterations in neurogenesis, neuronal maturation, neuronal connectivity and synaptic impairment as well as mitochondrial dysfunction and dysregulation of miRNAs in SZ patient-derived cultures compared to controls. We discuss the pros and cons of these models and describe the potential of using such models for deciphering the contribution of specific human neural cell types to the development of the disease.

## Introduction

Schizophrenia (SZ) is a complex, highly heritable psychiatric disorder. It affects 1–2% of the population world-wide and holds substantial health- and social care costs [24]. The onset is usually in late adolescence or early adulthood [133]. Psychopathology manifests as a mixture of positive symptoms such as delusions, hallucinations, confused thoughts and negative symptoms like lack of emotional responses, reduction in speech, social withdrawal and anhedonia. Additionally, patients have a high suicide rate [118] experience more physical illnesses [90] and reduced life expectancy by nearly 15 years [54].

Dysfunction in dopamine signalling is one of the most prominent hypotheses of SZ. Hyperactive dopamine transmission has been shown to be associated with psychosis in patients [1, 59], and dopamine D2-receptor blockers such as first generation antipsychotics-chlorpromazine and haloperidol are still widely used as treatments for SZ [79]. However, the dopamine hyperactivity hypothesis fails to explain core features such as negative and cognitive symptoms, which are not eased by dopamine antagonists. Furthermore, reduced dopamine release has been detected in the cortex and striatum of SZ patients [122, 141]. The discovery that glutamate NMDA receptor antagonists can induce SZ-like negative and cognitive symptoms in healthy subjets as well as worsen clinical manifestations in SZ patients has led to an alternative hypothesis about the involvement of glutamate signalling [72, 85]. This hypothesis claims that hypofunction of NMDA receptors can cause excessive glutamate release, thereby increasing cortical excitation. Post-mortem studies have shown that GABAergic interneurons are specifically affected by NMDA receptor dysfunction, as a reduction of NMDA subunit NR2A in interneurons leads to decreased expression of GABAergic-related transcripts such as glutamate decarboxylase 67 (GAD67) and parvalbumin (PV) [14, 164]. This reduction of GABAergic signalling may in turn cause disinhibition of the postsynaptic excitatory circuits. Building on this, animal models have revealed an imbalance of excitatory and inhibitory (E-I) activity in cortical circuits, including reduced activity of interneurons and GABAergic deficits in the prefrontal cortex [42, 135]. However, it remains unclear if this imbalance causes disease symptoms.

Today, the first line treatment of SZ is second-generation (atypical) antipsychotics, which only partially block dopamine receptors and have less side effects compared to traditional antipsychotics [44]. However, current treatments for SZ are only partially effective, alleviating at best psychotic symptoms while still causing considerable side effects, and approximately 30% of patients are classified as treatment resistant [58]. Notably, all available antipsychotic drugs are thought to work mainly through blockade of the type 2 dopaminergic receptor, and this main target has not changed since the discovery of this mechanism 60 years ago [21].

## Genetic risk factors of schizophrenia

The etiology of SZ is believed to be highly multifactorial, including both common and rare genetic variants as well as environmental factors [68]. The first formal genome-wide significant association of a single nucleotide polymorphism (SNP) was identified to the ZNF804A locus [105]. Subsequently, associations were also found to a large region in chromosome 6 corresponding to the major histocompatibility complex (MHC) and encompassing more than 500 genes [146]. Other SZ associated loci include dopamine D2 receptor (*DRD2*), glutamate receptor components (*GRM3, GRIN2A* and *GRIA1*) and serine racemase (*SRR*) [41, 124]. In 2018, a new GWAS study discovered 50 novel loci associated to schizophrenia and showed that common schizophrenia alleles are enriched in regions under background selection and mutation-intolerant genes [140].

A recent SNP study included 75.000 patients and identified 342 independent loci implicating 119 genes, which provides novel insights into the genetics of SZ [156]. The strength of such more high-powered genome-wide association studies (GWAS) is reflected in the derived SNP-based heritability in European ancestry cohorts of 0.24 (SE 0.007), i.e. roughly a quarter of the variance in liability can be attributed to the examined SNPs. Yet, these common variants are only responsible for a small proportion of the genetic contribution to schizophrenia (less than 5%) and vary in penetrance [156]. Other genomic studies have revealed several rare high risk variants of SZ including copy number variations (CNVs) 1q21.1, 3q29, 15q13.3, 16p11.2 and 22q11.2 [87, 123].

An exome sequencing study including almost 25,000 patients has further identified ultra-rare protein truncating mutations in 32 genes, most of which are implicated in the formation, structure and function of the synapses and are strongly associated with a risk of developing schizophrenia [139]. This discovery has pointed to synaptic dysfunction as a possible contributing cause of SZ. More specifically, the identification of ultra-rare variants in the NMDA receptor subunit GRIN2A and AMPA receptor subunit GRIA3 suggests a dysregulation of the glutamatergic system and of the formation of synapses to interneurons [139].

Importantly, GWAS also identified genes harbouring rare loss-of-function variants such as *STAG1*, *FAM120A*, glutamate receptor subunit *GRIN2A* and transcription factor *SP4* [139]. The fact that both GWAS and exome sequencing studies identified a group of genes involved in similar biological processes, such as pre-and post-synaptic processes in excitatory and inhibitory neurons, supports the convergence of common and rare variant associations in SZ. Although GWAS and whole exome sequencing are essential tools to understand SZ, they must be complemented by epidemiological studies to identify environmental components contributing to disease risk as well as by cellular and molecular studies on SZ models to unravel the causal relationships between the genotype–phenotype associations identified. In the following section, we will summaries the efforts done in developing animal models of SZ based on genes identified from GWAS studies. We will then dive into the advancements made in the field of using patient-derived or gene-edited human induced pluripotent stem cells (hiPSCs) to gain unique insights into the molecular and cellular pathways underlying SZ genotype–phenotype associations in a human neuronal context.

## Animal models of schizophrenia

Currently available animal models of SZ fall into three main categories: genetic, developmental and drug-induced. The first transgenic mouse model to be developed contained a dominant-negative form the the familial SZ gene Disrupted-in-schizophrenia 1 (*DISC1*), and showed impairments in neurons from prefrontal cortex and hippocampus- two regions implicated in SZ [52]. DISC1 plays a crucial role during neuronal development, however, DISC1 models are controversial, because they present only few SZ-like characteristics, but not complete phenotypes [69, 119]. Further, DISC1 is not found to be associated with SZ risk in more recent GWAS studies and its relevance to SZ is therefore questionable [131, 139]

Other genetic models are conditional knock-outs for neuregulin and its receptor ErbB4, showing distinct SZ-like characteristics. The Neuregulin/ErbB4 knockout mouse models exhibit positive symptoms, which disappear after administration of antipsychotic drugs [106, 157]. However, it should be noted that no single SNP in NRG1 has been identified as significantly associated to SZ across different patient populations [102, 146]. The 15q13.3 microdeletion mouse models reproduces symptoms such as long-term spatial memory impairment and auditory processing deficits, accompanied by neuronal hyperexcitability and reduced gamma oscillatory activity [36, 40]. Moreover, mice carrying the 15q13.3 microdeletion are more susceptible to peripubertal stress, leading to stronger SZ-related phenotype in adulthood.

The 22q11.2 deletion SZ mouse models exhibit dysfunction of cortical GABAergic interneurons, defects in synaptic transmission and impaired working memory [93]. A congenic 22q11.2 model was recently developed to avoid confounding effects of mutations in the background mouse strains. This model confirmed SZ-like characteristics such as prepulse inhibition (PPI) deficits and increased sensitivity to NMDA receptors and also showed an increase in the dopamine metabolite DOPAC in prefrontal cortex [32]. Mice carrying a 1q21.1 microdeletion also recapitulates key features of the dopamine hypothesis and is together with the 22q11.2 deletion model a new powerful tool to study dopamine alterations in SZ [99].

Maternal immune activation (MIA) in response to environmental factors like an infection, produces irreversible changes in CNS development and increases the risk for the unborn child to develop SZ later in life [67, 80, 100]. A mouse model using polyriboinosinic-polyribocytidilic acid (PolyIC) as an MIA-inducer similarly shows altered social interaction, cognitive decline and neurodevelopmental impairments in the affected offspring [27, 148]. Mouse models with NMDAR hypofunction were developed based on the observation that administration of NMDAR antagonists could lead to SZ symptoms in healthy subjects [72].

One of the most established models is a drug-induced model in which chronic administration of ketamine recreates numerous SZ-relevant phenotypes like interneuron impairment and altered cognition [35, 88]. In line with this, a rat model of apomorphine-induced SZ-like features also shows an imbalance of excitatory and inhibitory (E-I) activity in cortical circuits, including reduced activity of interneurons and GABAergic deficits in the prefrontal cortex [135].

These animal models are extremely valuable for investigating the potential underlying pathophysiology of SZ, however they are also hampered by challenges relating to species differences. The human cortex has gyrification, and contains > 1000-fold the number of neurons found in the mouse cortex [51]. Although the main neuronal cell types are relatively conserved between mammals, there are key differences in the cellular features of human neurons which are relevant for neuropsychiatric disorders [168]. In particular, the importance of the prefrontal cortex in human SZ can likely not be modelled in mice, where this structure is much smaller and less crucial for mouse behaviour [20]. As psychotic symptoms cannot be modelled in rodents, interpretation of the results from animal models should be carefully considered. Additionally, the interplay between the genetics and environmental factors, which could trigger SZ in humans is difficult to model in mice and currently there is no available animal model that mimics the complex etiology or polygenic background of SZ.

## Findings from hiPSC-based models of schizophrenia

A major limitation in understanding development of SZ is the gap existing in unravelling the causality between the many identified SZ risk genes and the pathology of the disease. Postmortem studies of patients have identified reduced neuronal size and spine density in the prefrontal cortex and hippocampus [163], and MRI scans reveal connectivity deficiencies, neurotransmitter dysfunctions, reduction of brain gray matter and an abnormal distribution of neurons in the prefrontal cortex [6, 138, 170]. However, postmortem tissue cannot be used to perform dynamic or interventional cellular studies on the pathways involved. Human induced pluripotent stem cells (hiPSCs) can be derived from any somatic tissue, including subject’s skin or blood cells, through reprogramming to the pluripotent state [149]. These derived hiPSCs can then in turn be used to generate cell types of the brain for in vitro.

Although most studies on hiPSC-derived SZ neurons studies about SZ have used 2-dimensional (2D) neuronal cultures, technical advances have lately allowed for generation of hiPSC-derived 3-dimensional (3D) brain organoids, which display self-organising capacities mimicking the anatomical structures of the early human fetal cortex [73]. Such 3D brain models can in some cases more accurately mimic the complexity of specific brain region development and cell interactions [34, 136].

**Fig. 1:**
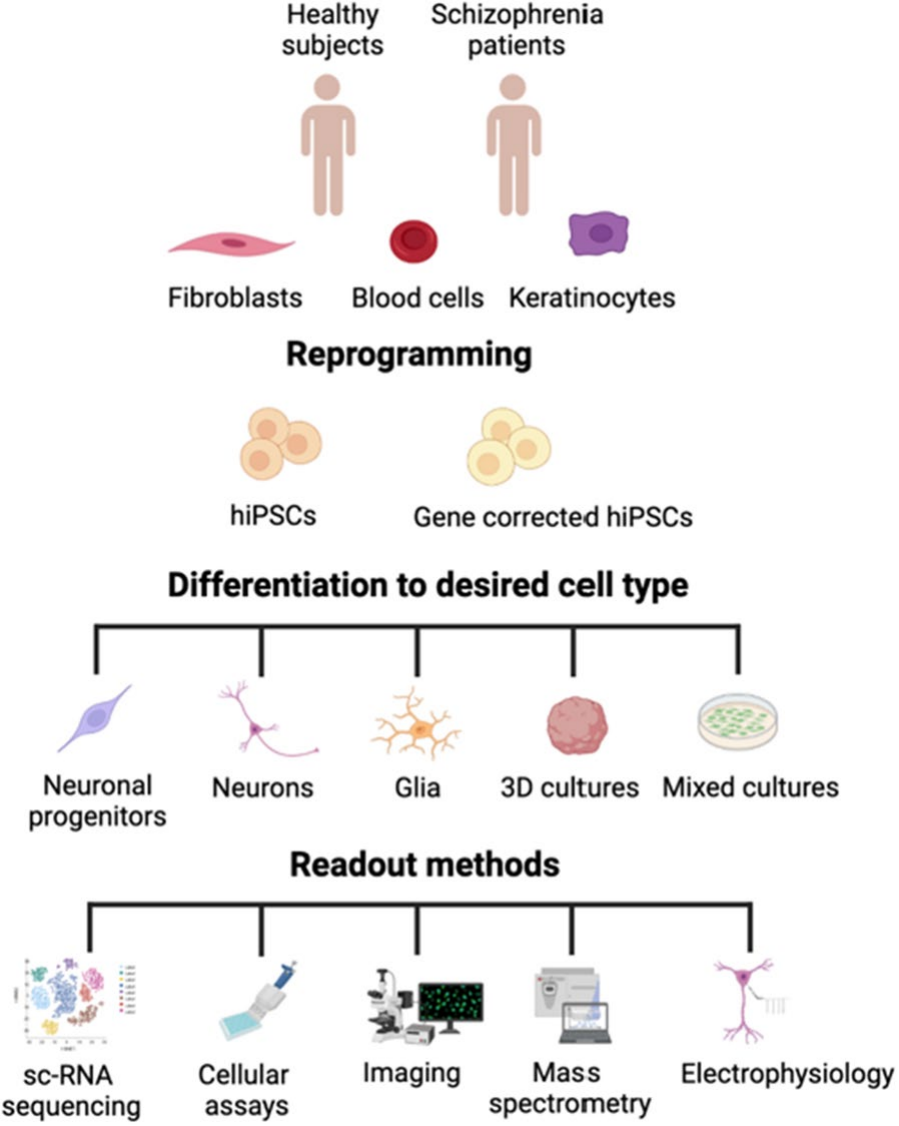
An overview of hiPSCs as a tool to investigate schizophrenia in vitro models. Fibroblasts, blood cells or keratinocytes from control subjects and/or schizophrenia patients are collected and reprogrammed to hiPSCs—some of which may be gene corrected and used as isogenic controls. The established hiPSCs can then be differentiated into the cells of interest, for example, neuronal progenitors cells (NPCs), neurons, glia or used for 3D or mixed cultures to model schizophrenia in vitro. Several readout methods such as single cell RNA (sc-RNA) sequencing, different cellular assays, cell imaging, mass spectrometry and electrophysiology can be used to investigate cell cultures in vitro

**Fig. 2:**
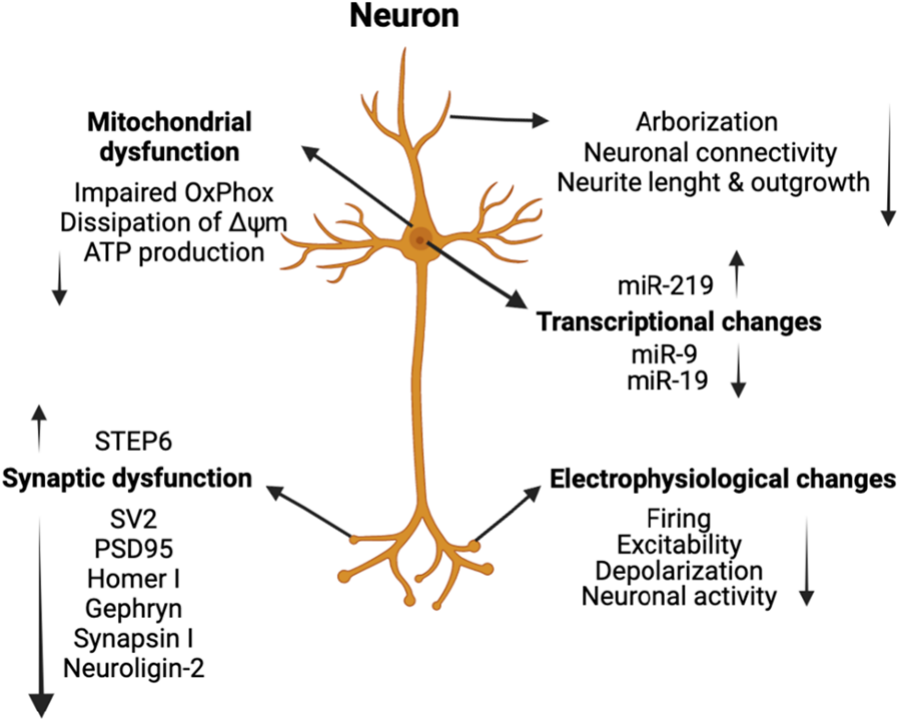
Schematic representation of key findings from studies on hiPSC-derived neurons. Neuronal defects found in hiPSC-based models of SZ can be characterized as reduced neuronal connectivity and neurite outgrowth, synaptic and mitochondrial dysfunction as well as neurodevelopmental and miRNA impairments. Additionally, changes in electrophysiology can be characterized as reduced neuronal firing, excitability, depolarization and overall neuronal activity

In this review, we systematically summarize the body of published work related to SZ-associated cellular phenotypes identified in hiPSC studies, and we find that the identified cellular mechanisms can be categorized into changes in neurogenesis, neuronal maturation, reduced neuronal connectivity, neurite outgrowth, synaptic and mitochondrial dysfunction and impairments of the glial cells (Figs. 1 and 2, Table 1). Below, we summarise the findings obtained from hiPSC-based SZ models into each of these categories, and we provide an overview in Table 1 of all the studies discussed.

**Table 1:**
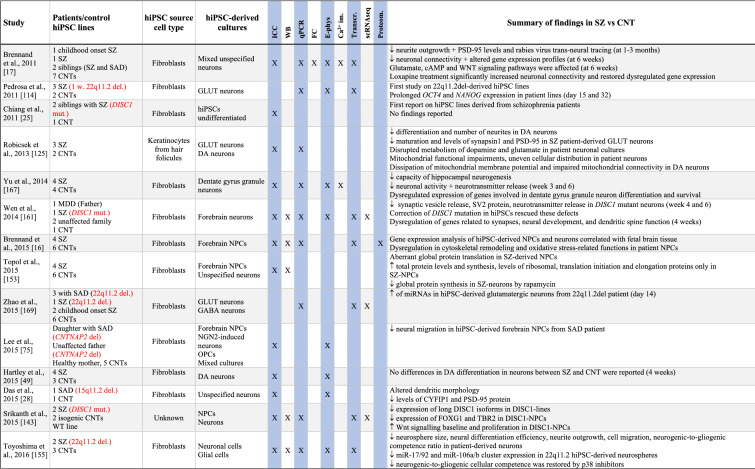

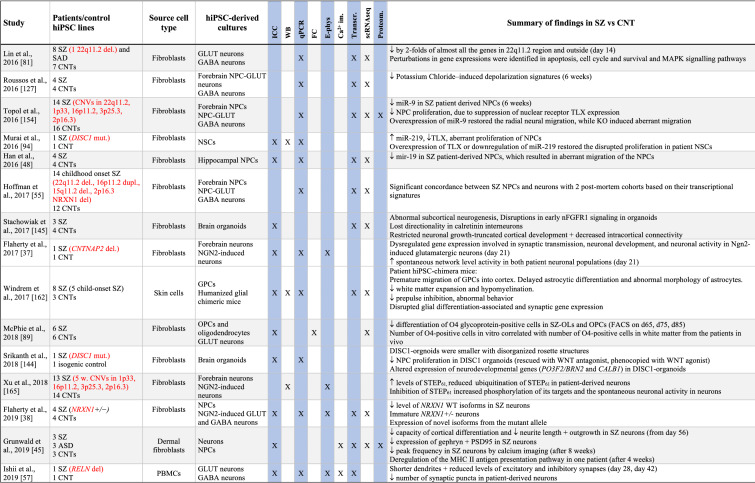

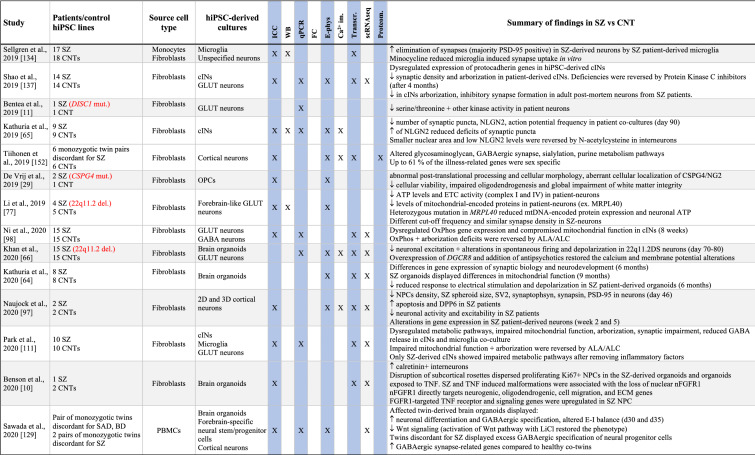

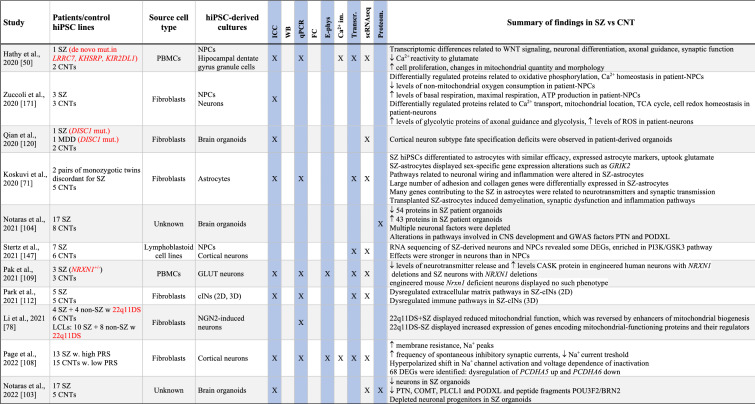
Summary of results from hiPSC-based schizophrenia studies

The table summarises published studies on hiPSC-based disease modelling of SZ, listed in chronological manner. Abbreviations and terms: ASD: autism spectrum disorder, Ca^2+^-im: Calcium imaging, cINs: cortical interneurons, CNT: healthy controls, CNV: copy number variation, DA: dopaminergic neurons, DEGs: differentially expressed genes, E-phys: electrophysiology, FC: flow cytometry, sequencing (or single nuclei RNA sequencing), GABA: GABAaergic (inhibitoryt), GLUT: glutamatergic (excitatory), GPCS: glial progenitor cells, hiPSCs: human induced pluripotent stem cell, ICC: immunocytochemistry, LCLs: lymphoblastic cells lines, MDD: major depressive disorder, NSCs: neural stem cells, NGN2: Neurogenin 2, NPCs: neural progenitor cells, OPCs: oligodendrocyte precursor cells, PRS: polygenic risk score, Proteom.: proteomics, SAD: schizoaffective disorder, scRNAseq: single cell RNA sequencing, SZ: schizophrenia, Transcr: transcriptomics (bulk RNA sequencing or microarray), WB: Western blotTranscr, ↑-increased, ↓-descreased

### Neurogenesis and neuronal maturation

An early study from 2011 showed reduced neuronal connectivity, decreased neurite number and altered gene expression in SZ-derived neurons [17]. Many components of glutamate, cAMP and WNT signaling pathways were affected and altered in these patient-derived neuronal cultures. Administration of the antipsychotic loxapine significantly increased neuronal connectivity and restored dysregulated gene expression [17]. Aberrations in hippocampal neurogenesis have been implicated in SZ pathology [47]. In line with this, reduced capacity of hippocampal neurogenesis from NPCs, lower levels of mature granule neuron markers *NEUROD1, PROX1 and TBR1* and reduced neuronal activity and neurotransmitter release has been observed in SZ patient-derived neurons [167].

Compromised differentiation into cortical neurons, significant reduction of neurite length, outgrowth and reduced calcium signaling in SZ patient-derived neurons has also been shown [45]. These findings are consistent with a study, where cellular models of 15 patients diagnosed with 22q11.2 deletion syndrome (22q11DS) revealed perturbed neuronal excitability, alterations in spontaneous firing and depolarization in 2D glutamatergic neurons and 3D organoids [66]. Further electrophysiological and imaging analyses showed an increase in excitability and impaired depolarization-induced L-type calcium channels (LTCC) calcium signaling in 22q11DS-derived neurons, which were related to a defect in the resting membrane potential (RMP) that caused voltage-dependent inactivation of calcium channels. Additionally, heterozygous loss of *DGCR8* was sufficient to recapitulate the functional defects observed in 22q11DS neurons. Likewise, 22q11.2DS defects could be rescued by overexpression of *DGCR8* and administration of antipsychotics, which restored the calcium and membrane potential alterations [66].

15q11.2 CNVs are leading risk factors for neuropsychiatric disorders, including SZ [86]. Neural precursor cells derived from 3 SZ patients hiPSCs with 15q11.2del mutation displayed NPC deficiencies in adherence junctions and apical polarity. The underlying reason was haploinsufficiency of *CYFIP1* located in 15q11.2 region, which encodes a subunit of the WAVE complex controlling actin cytoskeleton. Animal studies showed that deficiency in *Cyfip1* and WAVE signaling similarly affected radial glial cells leading to their ectopic localization outside of the ventricular zone in developing mouse cortex [166]. Interestingly, Cyfip1 interacts with Fmrp and cap protein eIF4E to regulate activity-dependent protein translation in mature neurons in mice [95]. Transcriptome analysis on 8 SZ patient-derived neurons with 22q11.2 DS showed twofold reduction in expression of almost all the genes in 22q11.2 region, including perturbations in gene expressions were found in apoptosis, cell cycle and survival and MAPK signaling pathways [81].

Contactin-associated protein-like 2 (*CNTNAP2*), a member of the neurexin family, functions as cell adhesion molecule, is associated with SZ [61, 126]. *Cntnap2* plays a role in axon guidance, dendritic arborization and synaptogenesis based on animal studies [3, 116]. Forebrain and NGN2-induced excitatory neurons were derived from 2 carriers of heterozygous intragenic *CNTNAP2* deletions, one affected and one unaffected [37]. *CNTNAP2* deletion affected expression of genes involved in synaptic transmission, neuronal development and neuronal activity in NGN2-induced glutamatergic neurons. Additionally, increased spontaneous network level activity in both patient neuronal populations were observed. These findings suggest that heterozygous *CNTNAP2* deletions may affect genes involved in neuronal development and activity. Furthermore, reduced neural migration was found in forebrain NPCs from schizoaffective disorder patient harboring deletion in *CNTNAP2*. This phenotype correlated with the exon and allele specific expression patterns of *CNTNAP2* in hiPSC-derived NPCs, neurons and oligodendrocyte precursor cells (OPCs) from one patient with *CNTNAP2* deletion [75]. Protein kinases phosphorylate proteins, regulate important pathways for synaptic transmission, plasticity, circuit formation and refinement during development [7, 43]. Therefore, dysregulation in kinase signaling might contribute to the synaptic impairment and is often associated with neurological and neuropsychiatric disorders [7, 26]. Investigation of hiPSC-derived cortical excitatory neurons from one SZ patient with a 4 bp mutation in *DISC1* showed global reduction of serine/threonine kinase activity, AMP-activated protein kinase (AMPK), extracellular signal-regulated kinase (ERK) and thousand-and-one amino acid (TAO) kinases [11]. This data supports a role of kinase impairment in SZ pathology and suggests kinases as a possible target for drug discovery.

Neurexins are the main regulators of neural circuits that control presynaptic release probability, postsynaptic receptor composition and synaptic plasticity [4]. In addition to that, *NRXN1* encodes the presynaptic cell-adhesion molecule neurexin-1 [87]. Among CNVs associated with SZ, 2p16.3 CNVs affect expression of *NRXN1* [87]. SZ patient-derived neurons with *NRXN1* deletions from 3 patients and engineered human neurons with *NRXN1* deletions displayed the same global decrease in neurotransmitter release and an increase in CASK protein, which is an intracellular *NRXN1*-binding protein. A distinct finding of this study was that engineered mouse *Nrxn1*-deficient neurons did not exhibit the same phenotype, which could imply a human-specific role for *NRXN1* [109]. Another study of SZ patient-derived glutamatergic and GABA-ergic neuron cultures with NRXN1 ± showed decreased levels of *NRXN1* WT isoforms and unexpected expression of novel isoforms from mutant allele in patient neurons. Besides, *NRXN1* ± neurons were not capable to fully mature [38]. These findings suggest a synaptic pathophysiological mechanism of SZ, where neurexins could be a target for new treatments.

### Neuronal connectivity and synaptic impairment

In the developing human brain cortex, synaptic density increases significantly until childhood whereas, during adolescence, the number of synapses gradually decreases until third decade of life [117]. Brain-specific tyrosine phosphatase, STEP (STriatal-Enriched protein tyrosine Phosphatase) together with STEP61, a membrane-associated phosphatase found in the postsynaptic density, are important regulators of synaptic function [15, 115]. It is known that STEP61 is elevated in postmortem brains of SZ patients, as well as in mice treated with psychotomimetics [23].

Forebrain neurons derived from 13 SZ patients (5 patients with childhood onset schizophrenia harboring CNVs) and 14 healthy subjects demonstrated increased levels STEP61 and reduced ubiquitination of STEP61 in patient-derived forebrain neurons [165]. Additionally, inhibition of STEP61 increased phosphorylation of STEP61 targets, which induced spontaneous neuronal activity in SZ neurons [165].

Reduced PSD95 density was observed in an early study with SZ patient-derived neurons [17]. These findings were consistent with results from a later study where lower levels of Synapsin I, PSD-95 and reduced number of synapses were observed in SZ-patient derived glutamatergic neurons [125]. Neuronal cultures derived from 3 SZ patients displayed a low expression of the inhibitory synapse marker gephryn and PSD95 [45]. Glutamatergic and GABAergic cultures derived from a *RELN* deletion SZ hiPSC line exhibited abnormalities in synapse formation in vitro [57]. Reduced numbers of Synapsin I and Homer I (presynaptic or postsynaptic markers for GABAergic neurons) and gephyrin (postsynaptic scaffolding protein in GABAergic synapses) were found. This data indicates overall reduced formation of excitatory as well as inhibitory synapses [57, 97]. GABAergic deficits in the prefrontal cortex are one of the major findings in post-mortem brain tissue, which indicates a decrease in the activity of cortical interneurons (cINs) [56]. In concordance with this, cINs derived from SZ hiPSCs showed lower levels of GAD67, gephryn and Neuroligin-2 (NLGN2). These findings were reproduced in co-cultures with excitatory neurons derived from 9 SZ patients and 9 healthy controls [65]. Interestingly, NLGN2 overexpression in SZ neurons rescued synaptic puncta deficits while NLGN2 knockdown in healthy neurons resulted in reduced synaptic puncta density [65]. In another study, increased membrane resistance and overall Na^+^ channel function was altered in cortical neurons derived from individuals with SZ [108].

A study of 14 SZ patient-derived cINs showed dysregulated expression of protocadherin genes [137]. Additionally, SZ patient-derived cINs exhibited reduced synaptic density and arborization. These deficiencies were reversed by Protein Kinase C inhibitors, which is a downstream kinase in the protocadherin pathway. Same phenotype was found in adult post-mortem brains from SZ patients and mouse models [137]. Alterations in glycosaminoglycan, GABAergic synapse, sialylation, purine metabolism pathways were identified in the cortical neurons derived from monozygotic twins discordant for SZ. Moreover, up to 61% of the illness-related genes were found to be sex specific [151]. A further study revealed increased levels of dipeptidyl peptidase-like protein 6 (DPP6), an accessory subunit of Kv4.2 voltage-gated potassium channels in SZ patient-derived cortical neurons, which caused reduction in neuronal activity [97].

Disrupted in Schizophrenia 1 (DISC1) is a protein encoded by *DISC1* gene in humans. Polymorphisms and deletions in this gene have been associated with different psychiatric conditions, such as bipolar disorder, autism, major depression and SZ [128, 150]. The study of family members, where a daughter was carrying a 4 Mb deletion in *DISC1* and diagnosed with SZ, displayed several synaptic abnormalities [161]. Mutant DISC1 reduced synaptic vesicle release, accompanied with lower levels of Synaptic vesicle (SV) protein 2 and lower frequency of excitatory spontaneous synaptic currents in SZ patient-derived forebrain neurons. Notably, mutant DISC1 dysregulated expression of genes related to synapses, nervous system development, dendritic spine function pathways and psychiatric disorders in human forebrain neurons [161]. Isogenic correction of *DISC1* mutation reversed these defects. Another group generated isogenic hiPSC lines with engineered mutations in exon 2 and 8 of the *DISC1*, leading *to* loss of long DISC1 isoforms and affecting NPC proliferation, baseline WNT signaling and the expression of NPC fate markers such as FOXG1 and TBR2 [143].

### Mitochondrial dysfunction

Mitochondria are involved in neuronal activity, important for synaptic function [9], Ca^2+^ signaling [46, 91], generation of action potentials [158] ion homeostasis [30, 53] and ATP synthesis. Neural cells derived from a SZ patient presented a two-fold increase in extramitochondrial oxygen consumption and increased levels of ROS. The elevated ROS levels were reverted by the mood stabilizer valproic acid (VPA) [113].

Mixed dopaminergic and glutamatergic neuron cultures derived from 3 SZ patients presented impaired differentiation capacity to dopaminergic cells and incomplete maturity of glutamatergic cells, accompanied by disrupted metabolism of dopamine and glutamate [125]. Morphological abnormalities were observed only in the dopaminergic cells, and mitochondrial functional impairments, uneven cellular distribution of organelles, dissipation of mitochondrial membrane potential (Δψ_m_) and perturbations in mitochondrial network structure and connectivity were found [125].

Significantly reduced ATP levels and reduced activity in oxidative phosphorylation complexes I and IV of the electron transport chain (ETC) in SZ patient-derived neurons with 22q11.2DS have also been observed [77]. Further, levels of protein products of mitochondrial-encoded genes such as MT-ND1 (complex I), cytochrome b (complex III), and COX1 (complex IV) were significantly reduced. One of the deleted genes in the 22q11.2 region is mitochondrial ribosomal protein L40 (MRPL40) [22], a SZ risk gene, which was reduced both in protein and mRNA levels in 22q11.2DS neurons [77]﻿. In support of this, transgenic mice lacking one copy of Mrpl40 show alterations in mitochondrial calcium and exhibited psychosis-related cognitive deficits [31]. A healthy control hiPSC with engineered heterozygous mutation of MRPL40 line revealed similar deficits in mitochondrial DNA-encoded proteins, ATP levels and complex I and IV activity, indicating that 22q11DS MRPL40 heterozygosity leads to reduced mitochondrial ATP production and altered mitochondrial protein expression.

Differentially regulated proteins in pathways related to mitochondrial function, oxidative phosphorylation, cell cycle control, DNA repair, Ca^2+^ homeostasis and neuritogenesis were observed in a study using hiPSCs from 3 non-familial SZ patients [171]. Metabolic analysis of patient-derived NSCs showed reduced levels of non-mitochondrial oxygen consumption, increased basal respiration and ATP production. Further, increased levels of glycolytic proteins of axonal guidance, glycolysis and ROS were identified in these SZ-neurons.

Likewise, mitochondrial impairments such as lower basal consumption rate, ATP production, proton leak, nonmitochondrial oxygen consumption, diminished response to stimulation and depolarization were observed in brain organoids from another study with non-familial SZ patient hiPSCs [64]. Analysis of gene expression revealed dysregulation of genes involved in mitochondrial function as well as modulation of E-I balance [64]. In support of this, reduced number and altered mitochondrial morphology were observed in NPCs and hippocampal DG granule cells from one SZ patient with de novo mutations in leucine-rich repeat containing 7 (*LRRC7*), K-homology type splicing regulatory protein (*KHSRP*), killer cell immunoglobulin-like receptor 2DL1 (*KIR2DL1*) [50].

Furthermore, a study with SZ patient-derived cINs showed dysregulated OxPhos related gene expression and compromised mitochondrial function, ultimately resulting in oxidative stress in the cells [98]. OxPhos deficit in cINs was reversed by Alpha Lipoic Acid/Acetyl-L-Carnitine (ALA/ALC).

A study with 8 individuals with 22q11DS (4 were SZ patients) showed that only neurons derived from the affected carriers (22q + SZ) had reduced ATP levels and OXPHOS activity. Neurons from unaffected individuals carrying 22q11DS (22q-SZ) had significantly upregulated genes, which encode OXPHOS subunits [78]. For instance, NDUFV2 expression was increased by 50% only in the 22q-SZ group. Additionally, expression of genes involved in mitochondrial biogenesis, for example PGC1α, showed a similar pattern of upregulation in the 22q-SZ group compared to the control and the 22q + SZ groups. In summary, several studies point towards impairments in oxidative phosphorylation and ATP production in neuronal in vitro models of SZ, and this may be a main underlying disease driver in 22q11DS patients.

### Developmental impairments mediated by miRNAs

MicroRNAs (miRNAs) are small non-coding RNAs that regulate gene expression by inhibiting translation or degrading RNA [8]. Strong evidence suggests that miRNAs, particularly miR-137, may contribute to the development of SZ [124]. Moreover, the coding region for a member of miRNA biogenesis, *DGCR8*, is located within 22q11.2, the most common SZ-associated CNV [63].

miR-19 expression modulates the migration and maturation of adult-borne neurons in the brain by suppressing Rap guanine nucleotide exchange factor 2 (Rapgef2) [48]. miR-19 was downregulated in SZ patient-derived NPCs and resulted in aberrant migration of the NPCs in the brain. Additionally, the aberrant expression of miR-19 inversely correlated with the expression of Rapgef2 [48]. These findings imply that dysregulation of miR-19a in the brain maybe also affects development of SZ.

Downregulation of miR-9 was observed in childhood onset SZ patient-derived NPCs from individuals carrying CNVs (22q11.2, 1p33, 16p11.2, 3p25.3, 2p16.3) [154]. Downregulation of miR-9 inhibited NPC proliferation by suppressing nuclear receptor TLX, which regulates NPC proliferation and self-renewal [121]. miR-9 effects are mediated by small changes in indirect miR-9 targets, rather than sizeable changes in direct miR-9 targets. Retroviral overexpression of miR-9 restored radial neural migration deficit in SZ-derived NPCs, whereas knockdown partially induced aberrant migration in control NPCs. Overall, reduced activity of miR-9 may contribute to the risk of developing SZ.

miR-219 is expressed in OPCs [83] and promotes oligodendrocyte differentiation by repressing negative regulators of this process [33]. miR-219 is amongst the most highly upregulated miRNAs in brain regions of SZ patients [13, 142]. In alignment with this, a study showed upregulation of miR-219, downregulation of TLX and impaired proliferation in DISC1-mutant hiPSC-derived NPCs [94]. Overexpression of TLX or downregulation of miR-219 rescued reduced proliferation in these cells. This study indicates that elevation of miR-219 expression reduces NSC proliferation in SZ patients.

### Implications of glial cells in schizophrenia

Over the past decade there has been accumulating evidence that glial cells may contribute to the pathogenesis of SZ [12]. During development, excessive activation of microglial cells, together with the genetic predisposition of the individual could collectively contribute to the development of SZ [74]. During human brain development a major function of microglia is synaptic pruning [130]. It was shown that excessive synaptic pruning by microglia contributes to the reduction in synapse density in SZ patients [19, 132]. Additionally, reduced synaptic density was observed in a postmortem adult brain from SZ patient [70]. Schizophrenia’s strongest genetic association at a population level involves variation in MHC locus, arising in part from many structurally diverse alleles of the complement component 4 (*C4*) genes [132]. These alleles generate highly varying levels of *C4A* and *C4B* expression in the brain, with each common *C4* allele associating with SZ in proportion to its tendency to generate greater expression of *C4A*. Human C4 protein localizes to neuronal synapses, dendrites, axons and cell bodies and is believed to be involved in synaptic pruning [132]. In line with this, increased elimination of synaptic structures has been observed by SZ patient hiPSC-derived microglia [134]. The majority of uptaken particles stained positive for PSD-95, and engulfment of particles was partly modulated by human SZ risk variants at *C4* locus [134], which is concordant with human postmortem adult brain studies [132]. Minocycline, a tetracycline with high brain penetrance, reduced synapse uptake in vitro in a dose-responsive fashion. Moreover, the reduction of psychosis associated with administration of minocycline in adolescents and young adults was detected by electronic health records (EHRs) [134]. These findings could suggest excessive pruning as a potential druggable target for treatment of SZ.

cINs, especially those expressing PV or somatostatin (SST), are strongly affected in individuals with SZ [76]. Altered cIN neurotransmission in SZ may account for abnormalities in gamma oscillations, which are associated with cognitive impairments in SZ patients [159]. Transcriptome analysis showed that SZ patient-derived cINs cocultured with activated microglia impaired metabolic pathways, compromised mitochondrial function, arborization, synapse formation and synaptic GABA release in cINs [111]. Deficits in mitochondrial function and arborization were successfully reversed by Alpha Lipoic Acid/Acetyl-L-Carnitine (ALA/ALC). Interestingly, only SZ-derived cINs cultures showed impaired metabolic pathways after removal of inflammatory factors.

Human astrocytes perform different roles in neurotransmitter release and uptake, supply neurons with substrates for energy metabolism, control extracellular water and electrolyte homeostasis in the brain, therefore they might be potential candidates for immune abnormalities in the development of SZ [160]. Human glial-mouse chimeras (i.e. mice transplanted glial cells from childhood-onset SZ patients) revealed several abnormalities such as premature migration of glial progenitors into mouse cortex, abnormal astrocytic morphology, delayed astrocytic differentiation and hypomyelination [162]. Additionally, elevated anxiety (elevated plus maze), sleep abnormalities (diurnal activity and sleep patterns test), reduced social interactions (three chamber test) in these mice were observed.

Along similar lines, a study on monozygotic twins discordant for SZ showed that patient hiPSC-derived astrocytes were able to differentiate with similar efficacy as control-astrocytes, expressed common astrocyte markers and performed glutamate uptake. However, aberrant expression of glutamatergic and GABAergic receptor genes in the SZ astrocytes was observed in a preprint study [71]. Glutamate receptor signaling appeared in the male but not female affected vs. unaffected twin comparison. In addition, SZ-astrocytes exhibited sex-specific gene expression alterations, which were deviated in Glutamate Ionotropic Receptor Kainate Type Subunit 2 (*GRIK2*). This gene was significantly upregulated in males but downregulated in females. Additionally, pathways related to neuronal wiring and inflammation were altered in SZ-astrocytes. Also, many adhesion and collagen genes were differentially expressed in SZ-astrocytes. This study demonstrated that expression of neural cell adhesion molecule L1-like protein (CHL1) between affected and unaffected males and females and between unaffected and healthy males was significantly altered. One of the functions of CHL1 is to regulate neuronal survival and growth and induce dendritic spine pruning in developing pyramidal neurons together with Semaphorin 3B. Astrocyte progenitors were transplated in mice brain to mature, which induced subtle behavioral changes in cognitive and olfactory functions and changes in gene expression in demyelination, synaptic dysfunction and inflammation pathways in mouse. All in all, this preprint study suggests a significant contribution of astrocytes to sex-specific risk in the development of SZ [71].

Another study found significantly reduced numbers of O4-positive cells from SZ patient lines, correlating to the reduction of white matter in the same individuals, as assessed by MRI [89]. A complimentary study investigated oligodendrogenesis in the context of familial SZ from siblings with missense mutations in the Chondroitin sulfate proteoglycan 4 (CSPG4) gene [29]. OPCs derived from CSPG4A131T carriers had impaired post-translational processing, subcellular localization of the mutant NG2 protein, aberrant cellular morphology and decreased cell viability and myelination capacity. This was in alignment with clinical findings showing impaired global white matter integrity in patients by diffusion tensor imaging [29]. Altogether, these results point that dysfunction in OPC development can be a significant player in SZ development.

### Disease modelling using brain organoids

3D models or brain organoids can be derived from hiPSCs or hESCs and provide the opportunity to better model complex structures of the human brain such as ventricular zone neurogenesis, neural circuits and regional connectivity [39, 60, 84]. A study looking at neurogenesis in brain organoids found abnormal dispersion of SZ patient-derived NPCs from the ventricular zone (VZ) into the intermediate (IZ) and cortical zones (CZ) in SZ patient-derived cultures compared to controls [145]. This study further identified restricted neuronal growth, resulting in truncated cortical development and decreased intracortical connectivity. The decreased intracortical connectivity was specified by changes in the orientation and morphology of calretinin positive cINs. This evidence suggests that SZ might be programmed at the preneuronal stage and involves a common mechanism of dysregulated Integrative Nuclear FGFR1 (nFGFR1) signaling. nFGFR1 binds to the promoters of genes that control the transition from proliferation to cell differentiation and to the morphogens that delineate the body and CNS axes, construct the nervous system [96]. Consistent with earlier reports, nFGFR1 signaling was dysregulated in SZ patient-derived brain organoids, contributing to abnormalities in the cortical architecture [96, 145].

In line with this, brain organoids with DISC1 mutation showed reduced size, disorganized rosette structure and reduced NPCs proliferation. This phenotype was phenocopied by WNT agonism and rescued by WNT antagonism. Moreover, this study revealed alterations in expression of genes important to neuronal development, like POU3F2/BRN2 and CALB1 [144]. DISC1 mutation effects were also studied in sliced human brain organoids, where the mutation was found to cause a loss of proper layer formation [120]. In addition to that, deficits of impaired laminar distribution and increased marker co-expression phenocopied control SNOs treated with the β-catenin antagonist IWR, indicating a possible role of WNT signaling in the pathogenesis of SZ. However, the relevance of DISC1 mutation in SZ pathology is debatable, as this gene does not appear to be a clear genetic risk factor for SZ in newer GWAS or exome sequencing studies.

A study applying brain organoids and forebrain NPCs from pairs of monozygotic twins discordant for SZ found increased neuronal differentiation, GABAergic specification and altered E-I balance in the brain organoids from the affected twin [129]. Interestingly, this also involved a reduced WNT signaling which could be restored after treatment with LiCl. Two monozygotic twin pairs discordant for SZ displayed excess GABAergic specification of their NPCs, which was followed by increased expression levels of GABAergic synapse-related genes. This study suggests that altered E-I balance during brain development might underlie the development of SZ.

As previously discussed, activation of prenatal immunity might contribute to SZ pathogenesis, due to secretion of inflammatory factors such as tumor necrosis factor-α (TNF-α). SZ patient brain organoids exposed to the cytokine TNF, led to abnormal dispersion of NPCs through VZ and CZ, which is concordant with phenotypes from Stachowiak et al., in 2017 [10]. Additionally, loss in cellular compostion and disorientation of cINs were observed in SZ patient-derived organoids and organoids exposed to TNF. Both SZ- and TNF-induced malformations were associated with the loss of nuclear nFGFR1 form in the CZ and its upregulation in deep IZ regions. Evidently, 3D models provide the opportunity to investigate molecular mechanisms for TNF-dependent neurodevelopmental pathology of SZ, its connections to maternal infections and elevated immunity during prenatal development. Moreover, transcriptomic analysis showed dysregulation in extracellular matrix pathways in SZ patient-derived migrating cINs, whereas sphere cINs from SZ patients showed dysregulation in immune pathways with HLA genes being mostly affected [112]. A transcriptional analysis of SZ patient hiPSC-derived brain organoids revealed downregulation of multiple neuronal factors such as MAP2, TUBB3, SV2A, GAP43, CRABP1, NCAM1 [103]. This study further identified prominent alterations in neurodevelopmental factors such as COMT, PLCL1 and POU-domain fragments POU3F2/BRN2, which were downregulated, as well as altered expression of novel GWAS factors, Pleiotrophin (PTN) and Podocalyxin (PODXL) in patient organoids. SZ patient-derived brain organoids showed lower NPCs survival, which led to formation of fewer neurons [103]. This study further identified that transcription factor BRN2 and growth factor PTN operate as mechanistic substrates of neurogenesis and cellular survival in patient organoids.

## Discussion

### Main mechanistic insights from hiPSC-based studies

Despite extensive efforts in performing GWAS analyses in hundreds of thousands of SZ patients, the underlying cause of the disease is still largely unknown. Studies of adult postmortem brain tissue from SZ patients and animal models have provided substantial knowledge on the pathology of the disease. However, these approaches have significant limitations, as animal models do not recapitulate the polygenic nature of the disease, and post-mortem studies may be hampered by delays in tissue preservation which interfere with RNA, DNA and protein preservation, making molecular and biochemical studies biased. Additionally, post-mortem tissues do not allow for dynamic molecular and cellular intervention studies.

hiPSCs allow for derivation of different brain specific cell types such as cortical neurons, microglia and interneurons, thereby yielding the opportunity to create SZ patient-specific disease models to investigate cellular and molecular phenotypes [2, 92, 101]. The use of SZ-derived hiPSC models have overall converged on identifying impairment in neuronal maturation and reduction of neurite length and outgrowth as one of the hallmarks of SZ-derived cells [17, 45, 57, 66, 75, 125]. Another hallmark identified in several studies was an E-I imbalance, including increased neuronal specification into GABAergic neurons and upregulation of genes, related to GABAergic neurotransmission [66, 111, 129]. This is in concordance with the E-I imbalance hypothesis generated from clinical findings of SZ patients [82, 107]. Synaptic impairments and overall reduction in the formation of excitatory and inhibitory synapses were also identified in several studies, including reduced expression of synaptic proteins such as PSD95, Synapsin I and Gephryn [17, 45, 57, 65, 97, 125]. Likewise, mitochondrial functional impairments, uneven cellular distribution of organelles, dissipation of mitochondrial membrane potential (Δψ_m_), reduced ATP and perturbations in mitochondrial network structure and connectivity were identified by different groups [31, 50, 64, 77, 125]. Strong evidence suggests that miRNAs are strongly involved in the development of SZ [124], and in line with this, several studies have shown dysregulation of miRNAs in hiPSC models of SZ [48, 94, 121, 154]. Additionally, accumulating evidence showed that glial cells may contribute to the pathogenesis of SZ [12], and dysfunctional microglia and astrocytes were highlighted in various articles [19, 71, 111, 132, 134]

3D models or organoids display the spatial organization of the neural and glial brain cells, which gives the chance to investigate cell–cell interactions, therefore it is useful tool to investigate development of SZ [10, 104, 145]. However, a limitation of organoids is batch to batch variability in cellular composition, size of the structure and complications of a necrotic core. This could potentially be addressed by 3D printing or by developing vascular systems in organoids [18, 57] and 3D printing.

### Are we studying the right cell types?

Recent studies from different research groups have integrated single-cell RNA sequencing (scRNAseq) data with GWAS data from large SZ cohorts to identify the cell types with the highest enrichment for expression of SZ-associated risk genes. An initial 2018 study by Skene et al. cross-referenced data from two large-scale SZ GWAS studies [110, 124] with scRNAseq data from the adult mouse brain, and they found significant enrichment for SZ risk genes in hippocampal CA1 pyramidal neurons, medium spiny neurons (MSNs), striatal PV-expressing interneurons and somatosensory pyramidal neurons from cortical layers 2/3, 4, 5 and 6 [140]. A recent study of 76,755 people with SZ and 243,649 controls reported common variant associations at 287 distinct loci and in concordance with the Skene et al. study found that variant genes were significantly enriched in exactly the same cell types: hippocampal CA1 pyramidal cells, MSNs, somatosensory pyramidal neurons and interneurons [156]

This latter study further concluded that there were no genetic associations to microglia, astrocytes or any non-neural tissues, and that the SZ risk genes were primarily implicated in processes related to neuronal function, particularly synaptic organisation, differentiation and transmission [156]. These studies thereby converge on identifying the 4 specific neuronal subtypes mentioned above as likely being the main culprits in the development of SZ. It should be noted however that certain neuronal subtypes which may not be well-represented in the underlying scRNAseq datasets would fail to be identified by this method.

While most hiPSC studies performed to date have been applying cortical-type neurons, a few have also studied interneurons, hippocampal neurons and dopaminergic neurons (Table 1). For more relevant insights into SZ pathology, it would be relevant to focus future hiPSC studies on these latter cell types while also studying the MSNs, which have not yet been investigated in hiPSC-based studies.

### Pros and cons of hiPSC-based models

Using hiPSCs to model SZ has many advantages. One is the possibility to model SZ while preserving the genetic background of the patient, which provides the opportunity to use these models for studying not only monogenetic or CNV forms of SZ, but also the more common polygenic forms of the disease. Secondly, hiPSC models allow for performing dynamic studies on pathogenic pathways and receptors in the context of authentic human neurons, thereby avoiding risk of being misled by rodent-specific cellular characteristics. Direct downstream assays can be done using cellular readouts such as electrophysiology, morphological examinations and transcriptomic and proteomic analyses, which might reveal pathology at different time points and in different cell types. A particular advantage of performing disease modelling in hiPSCs is the ability to recapitulate human developmental neurogenesis and neuronal maturation in vitro, thereby allowing to investigate early timepoints of pathology on a molecular basis. Further, the polygenic background of SZ can be modelled in hiPSC, but cannot be modelled in animals, as these are usually based on maniplations of single genes or CNVs with large effect size. On the other hand, there are several limitations of modelling SZ with hiPSCs. In particular, higher brain function and complex cognitive and psychosocial impairments found as primary manifesting symptoms in patients cannot be modelled in cellular systems. Furthermore, hiPSC-derived models are heterogeneous, and may at times be hard to reproduce, as they are highly dependent on culturing conditions, cell lines, differentiation protocols and freezing–thawing cycles of the cultures. In older, less optimised protocols for hiPSC generation, the donor and cell source used for reprogramming could also induce variation in the differentiation capacity of the derived hiPSCs [62]. Moreover, hiPSC cultures mainly produce immature fetal-like neurons, which limits proper modelling of adult brain pathology. Therefore, it is important to apply optimized and reproducible cell differentiation protocols for disease modelling studies, and thorough subtype characterization of the cultures must be done to establish which cell types are present. Additionally, hiPSC disease modelling studies should employ stricter quality control measures to standardize culture composition, and each study should include equal amount of disease and control cell lines. A large meta-analysis of hiPSC genetic abnormalities reported in more than 100 publications identified 738 recurrent genetic abnormalities [5]. Therefore, karyotyping and oncogene testing should be routinally performed to ensure genetic intergrity of the cells.

GWAS data combined with scRNASeq data suggest that key neuronal subtypes are implicated in SZ etiology [140]. Future hiPSC studies should further investigate these specific subtypes in advanced cellular models such as defined regionalized brain organoids or cultures with controlled populations of mixed cells such a cortical excitatory neurons combined with specific populations of interneurons and MSNs to decipher the interactions between SZ risk genes and each specific cell types. To summarize, identification of key cell types implicated into SZ pathology through hiPSC studies can lead to a deeper understanding of the molecular mechanisms behind the genetic background of SZ and thereby potentially to the development of new treatments.

## Data Availability

Not applicable.
